# Estrogenic potency of bisphenol S, polyethersulfone and their metabolites generated by the rat liver S9 fractions on a MVLN cell using a luciferase reporter gene assay

**DOI:** 10.1186/1477-7827-12-102

**Published:** 2014-11-04

**Authors:** Jae Soon Kang, Jin-Soo Choi, Woo-Keun Kim, Yong-Ju Lee, June-Woo Park

**Affiliations:** Gyeongnam Department of Environmental Toxicology and Chemistry, Korea Institute of Toxicology, Jin-Ju, Gyeongnam Republic of Korea

**Keywords:** Endocrine disruptor, Bisphenol A, Bisphenol S, Polyethersulfone, MVLN cell, Estrogenic activity, S9 fraction, Metabolism

## Abstract

**Background:**

Bisphenol A (BPA) is an applied chemical that is used in many industrial fields and is a potential endocrine disruption chemical (EDC) that is found in the environment. Bisphenol S (BPS) and polyethersulfone (PES) have been suggested as putative BPA alternatives. In this study, the estrogenic potency induced by the binding of 17-beta-estradiol (E2), BPA, BPS, PES and their metabolites formed by the rat liver S9 fraction to the human estrogen receptor (ER) was estimated.

**Methods:**

We used an *in vitro* bioassay based on the luciferase reporter assay in MVLN cells to evaluate the estrogenic activity of 17-beta-estradiol (E2), BPA, BPS, PES (E2: 0.001 to 0.3 nM; BPA, BPS and PES: 0.0001 to 5 microM) and their metabolites (E2: 0.05 microM; BPA, BPS and PES: 0.1 mM) according to incubation times (0, 20 and 40 min). After chemical treatment to MVLN cells for 72 hrs, and the cell viability and luciferase intensity induced were estimated, from which the estrogenic activity of the chemicals tested was evaluated.

**Results:**

BPA and BPS induced estrogenic activity whereas PES did not show any estrogenic activity in the concentrations tested. In an *in vitro* assay of metabolites, BPA metabolites displayed comparable estrogenic activity with BPA and metabolites of both BPS and PES showed increasing estrogenic activity.

**Conclusions:**

The results suggest that the metabolites of BPS and PES have estrogenic potential and the need for the assessment of both chemicals and their metabolites in other EDC evaluation studies. The estrogenic potency of PES and its metabolites is the first report in our best knowledge.

**Electronic supplementary material:**

The online version of this article (doi:10.1186/1477-7827-12-102) contains supplementary material, which is available to authorized users.

## Background

According to the Environmental Working Group (http://www.ewa.org), numerous chemicals have been produced and used in the industrial, medical and agricultural areas, and an average of 2,000 novel chemicals are registered annually in the USA. There is a growing concern over synthetic substances that have the potential to interfere with endocrine systems and that subsequently impact the maintenance of homeostasis, reproduction, development and/or behavior in organisms by mimicking or antagonizing the biological functions of natural hormones [1]. These are commonly called endocrine disruption chemicals (EDCs). The World Health Organization (WHO) defines an EDC as an exogenous substance or mixture that alters the function of the endocrine system and consequently causes adverse health effects in an intact organism, or its progeny, or (sub) populations [2]. The group of EDCs is various and heterogeneous and includes industrial materials and their by-products: dioxin, bisphenol A (BPA), persistent organic pollutants (POPs), phthalates, pharmaceutical agents and pesticides [3].

BPA, well-known EDC, has been widely used as a material for the production of epoxy resins, phenol resins, and polycarbonate plastics; as an antioxidant in PVC plastics for the packaging of foods (lacquered coating of food cans) and drinks (polycarbonate bottles); and as a thermal paper coating [4, 5]. Some previous studies proved that BPA was toxic to endocrine functions in organisms by disrupting the ligand-activated estrogen receptor (ER)-mediated estrogenic activation [6–9]. Besides, BPA was reported to increase the testosterone secretion and inhibit the cancer resistance protein in the human and murine cells [10]. Because of the potent endocrine toxicity of BPA, the US Food and Drug Administration (FDA) warned of the possible hazards of BPA to fetuses, infants and children [11], and many countries, including the US, the members of the EU and Canada, have restricted its use in products.

Due to the growing body of evidence on BPA toxicity, there have been extensive efforts to find and replace BPA with materials that are equally effective in its function but that are less toxic. Subsequently, bisphenol S (BPS), tritan, polyethersulfone (PES) and polyethylene terephthalate (PET) were proposed and considered to be safe alternatives for BPA. BPS, a BPA analog, has been broadly applied in the manufacture of resins and plastics because it is stable at high temperatures and more resistant to sunlight and biodegradation compared to BPA [12, 13]. Additionally, PES is currently used in industrial resins and plastics because of its physical stability at temperatures as high as 200°C [14]. However, their environmental suitability (i.e., less endocrine toxicity) to replace BPA used in products has not been thoroughly investigated. Recently, some studies have reported that BPS had a comparable weak estrogenic potency compared with BPA, which in turn could disrupt E2-induced cell signaling, leading to altered cell proliferation, cell death, and prolactin release [13, 15–17]. To the best of our knowledge, there has been no study that evaluated the potential estrogenic activity of PES; however, this chemical (or metabolites) is likely to be estrogenic because it is a polymer form of BPS [18, 19]. Therefore, the degraded form or metabolites of PES by physical and chemical reactions is likely to have a similar structure to BPS.

Most chemicals are metabolized once absorbed into the body, and some metabolites may have different chemical features and toxic potency from the parent chemical [20]. BPA metabolites had a potent estrogenic activity [17, 21] and have been shown to enhance or decrease estrogenic activity according to the metabolic pathways affected when compared with BPA [20]. BPS metabolites produced by a rat liver S9 fraction had a weak estrogenic activity in transgenic yeast when measured by β-galactosidase [22]. Grignard et al. suggested that the metabolites of BPS and BPA might show different estrogenic activity with respect to their respective parent chemicals, [15] implying the necessity of investigating endocrine disrupting capacity between the metabolites as well as the parent chemicals, which could help us to understand the comprehensive environmental suitability of the chemicals as BPA alternatives.

In the present study, we estimated the estrogenic activities of BPA alternatives, BPS and PES, using a transgenic MVLN cell line that had a luciferase gene as the reporter gene. Moreover, we assessed time-dependent changes in the estrogenic activity of the metabolites of the two BPA alternatives. Finally, we discussed the suitability of BPS and PES as BPA alternatives.

## Methods

### Chemicals

**Figure 1:**
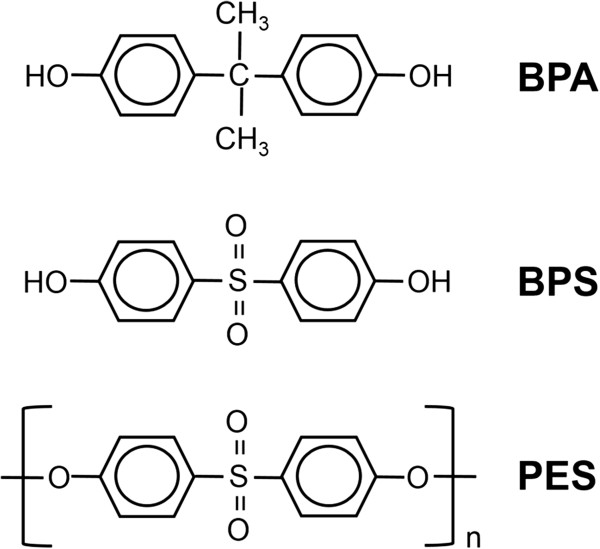
**The structures of BPA, BPS and PES.** BPA and BPS are bisphenol analogues that have two hydroxyphenyl functionalities. PES is a polymer of BPS.

(17β)-estra-1,3,5(10)-triene-3,17-dio (17-β-estradiol, E2, CAS number: 50-28-2, ≥98%), 2,2-bis (4-hydroxyphenyl) propane (BPA, CAS number: 80-05-7, ≥99%) and 4,4'-sulfonyldiphenol (BPS, CAS number: 80-09-1, 98%) were purchased from Sigma-Aldrich (St Louis, MO, USA), and polyethersulfone (PES, CAS number: 25608-6-3, 100%) was purchased from Goodfellow Cambridge Limited (Cambridgeshire, UK). The structures of BPA, BPS and PES were shown at Figure 1. All of the test chemicals were dissolved in dimethyl sulfoxide (DMSO; Junsei Chemical, Tokyo, Japan).

### MVLN cells

The MVLN cell line used in this study was kindly donated from Dr. Kyungho Choi at Seoul National University (Seoul, Republic of Korea). The MVLN cell line was a stably transfected MCF-7 cell line, a human breast carcinoma cell line, with a luciferase reporter gene plasmid consisting of a *Xenopus laevis* vitellogenin promoter region, which contains four estrogen responsive elements with the herpes simplex thymidine kinase promoter upstream of the firefly luciferase reporter gene [23, 24]. MVLN cells were cultured in Dulbecco’s Modified Eagle’s media: Ham’s F12 nutrient mixture without phenol red (Sigma-Aldrich, St Louis, MO, USA) containing sodium bicarbonate (Amresco, Solon, Ohio, USA), 10% FBS (Hyclone, Logan, UT, USA), 1% Sodium pyruvate (Sigma-Aldrich), 0.5 ml of insulin (1 mg/ml, Sigma-Aldrich) and 1% (v/v) antibiotic solution (penicillin and streptomycin, Invitrogen, Carlsbad, CA, USA). The cells were incubated at 37°C in a 5% humidified CO_2_ incubator (Thermo Fisher Scientific, Waltham, MA, USA).

### ER luciferase reporter gene assay

The ER binding assay is referred to in the OECD guideline for the testing of chemicals (OECD TG455, 2009) and the previous study [25]. MVLN cells were prepared in the assay medium (phenol red-free DMEM/F12 containing 10% charcoal-dextran treated FBS (Hyclone)). The 5 × 10^4^ cells in 250 μl of assay media were seeded on a 96-well white, clear bottom plate and were incubated for 24 h at 37°C. After the 24 h incubation, serially diluted stocks (30, 15, 7.5, 3.8, 1.9 and 0.1 nM of E2, and 500, 50, 5, 1, 0.1, and 0.01 μM of tested chemicals) were dissolved in DMSO to generate final concentrations, and chemicals were prepared. Each chemical stock solution (2.5 μl of each) was used to treat 250 μl of cells (final DMSO 1%, v/v): Final concentrations of tested chemicals, 0.3, 0.15, 0.075, 0.038, 0.019 and 0.001 nM of E2, and 5, 0.5, 0.05, 0.01, 0.001, and 0.0001 μM of BPA, BPS and PES. The chemical-treated cells were incubated at 37°C for 72 h in a 5% CO_2_ incubator. Twenty microliters of 5× CellTiter-Fluor™ Reagent (Promega, Madison, WI, USA) was added to all of the tested cells, and the mixtures were briefly mixed by orbital shaking at 500 rpm for 30 s. After incubation for 30 min at 37°C, cell viability was measured by Synergy H1 microplate reader (Bio-Tek, Winooski, VT, USA) at 380 nm (Ex) and 505 nm (Em). To evaluate the luciferase activity, 100 μl of One-Glo™ Reagent (Promega) was added to all of the tested cells, and the mixtures were incubated for 3 min at room temperature. The luminescence intensity was measured by Synergy H1 microplate reader (Biotek). All treatments were independently triplicated and each treatment was performed at three repeats.

### Estimation of ER gene expression using quantitative real-time PCR (qPCR)

To estimate the effect of ER expression by BPA, BPS and PES, three chemicals (each 0.1 and 0.5 μM finally) were treated with 1.25 × 10^5^ cells on 6-well plate and incubated at 37°C for 72 h in a 5% CO_2_ incubator. After cell harvest, total RNA was extracted from the cells using RNeasy mini kit (Qiagen, Hilden, Germany) and cDNA was synthesized using SuperScript III First-Strand Synthesis System for RT-PCR (Invitrogen, Carlsbad, CA, USA) according to manufacturer’s procedure.

The qPCR mixture (20 μl) was composed of 1 μl forward primer (5 μM), 1 μl reverse primer (5 μM), 5 μl cDNA (4 ng/μl), 10 μl 2× qPCR GO taq master mix (Promega) and 3 μl sterile water. The qPCR was performed at Agilent Mx3005P qPCR system (Agilent Technologies, Wilmington, DE) as follows: 10 min at 95°C (1 cycle), 20 sec at 95°C, 20 sec at 58°C and 20 sec at 72°C for 40 cycles, followed by a dissociation step for 10 sec at 95°C, 10 sec at 55°C and 10 sec at 95°C for 1 cycle. The primers of ER and β-actin as reference gene were referred from Kurebayashi et al. and Al-Bader et al. [25, 26]. Relative transcriptional level was estimated by 2∆∆Ct method [27]. The PCR efficiency was determined on 90–110% under optimized qPCR conditions and the product specificity was checked by the dissociation curve analysis. All experiments were triplicated. The transcriptional levels among treatments were statistically analyzed by one-way analysis of variance (ANOVA) followed by the post hoc comparison, Least Significant Difference (LSD) test using the SPSS software (SPSS v20, IBM, Armonk, NY, USA) (*p* <0.05).

### Incubation of chemicals with S9 fraction

To evaluate the estrogenic activities of the metabolites of BPA, BPS and E2, their respective parent chemical was incubated with a rat liver S9 fraction (Gibco, Carlsbad, CA, USA) according to the method described in the previous study [22]. Each 0.1 M parent compound (but E2 is 0.05 μM) was incubated in 80 μl of 0.1 M potassium phosphate buffer (pH 7.4) containing 0.25 mg/ml of the rat liver S9 fraction and 1% DMSO at 37°C for 5 min. The reaction was initiated by adding 20 μl of a NADPH-generating system (BD Gentest, Franklin Lakes, NJ, USA) to the final 100 μl reaction volume and was incubated, with shaking, at 37°C for 40 min. Each reaction mixture without the 40 min shaking-incubation after adding acetonitrile was used as a negative control (inactivated S9). The quenched reaction mixtures that were generated by the addition of 50 μl of acetonitrile were placed on ice for 15 min and were centrifuged at 18,000 × g for 5 min at 4°C. The supernatants were transferred to new tubes and completely evaporated using Scanspeed 32 (Labogene Aps, Copenhagen, Denmark). The extracts were dissolved in 50 μl of DMSO. Each sample solution (2.5 μl) was applied for assessment by the estrogenic activity assay.

### Estrogenic activity of PES revealed in acetonitrile

PES is known to attack by some organic solvents such as acetone and acetonitrile. In the metabolism procedure, because acetonitrile was used to stop the metabolism, there was the possibility that the product generated by attacking of solvent induced the estrogenic activity. Thus, we tried to estimate the estrogenic potency of PES treated on acetonitrile. PES was treated with or without 50 μl of acetonitrile for 15 min in ice. The estrogenic activity of PES treated with or without acetonitrile was performed by the procedure described above.

### Data and statistical analysis

The estrogenic activity of the tested chemicals was calculated by comparing each luciferase activity obtained as the mean relative luminescence units (RLU) to that of the solvent control. The effective concentration (EC) values and relative potency (REP) were calculated by the method referred to by Villeneuve et al. [28]. To estimate the EC values and REPs of the tested chemicals with respect to estrogenic activity of E2, a standard curve of each test chemical was constructed by using normalized luciferase units (i.e., RLUs of test chemical – RLUs of solvent control) and converting these values to a percentage of the mean maximum response of E2. Differences among each chemical was analyzed by one-way analysis of variance (ANOVA), followed by the post hoc comparison Least Significant Difference (LSD) test using the SPSS software (SPSS v20, IBM, Armonk, NY, USA) and a *t*-test using the Sigma Plot software (Systat Software, Chicago, IL, USA). Differences were considered statistically significant at *p* <0.05. The data represent the mean values of three independent experiments that were performed in triplicate.

## Results

### Estrogenic activities of tested chemicals

**Figure 2:**
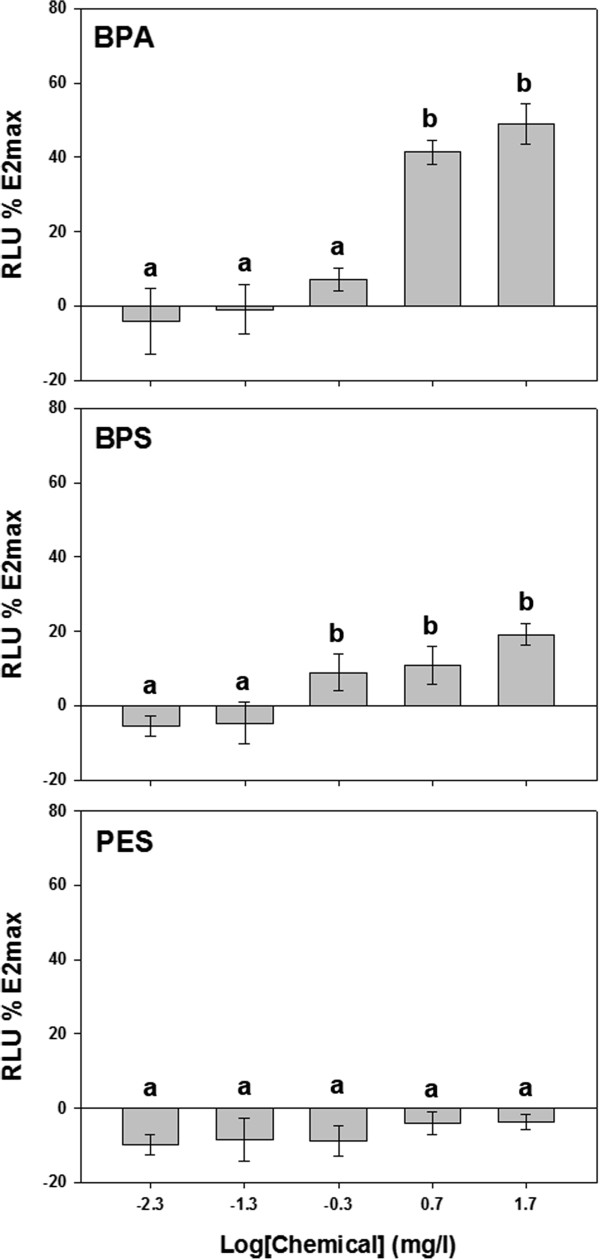
**The luciferase activity of BPA, BPS and PES according to the exposure concentrations.** The activity ratio was measured by compare to the luciferase activity of the highest concentration (E2max). Data represent the mean ± SD were statistically analyzed by ANOVA followed by the Least Significant Difference (LSD) test (p <0.05). All tests were performed in triplicate.

From the several concentrations of E2, BPA, BPS and PES, the luciferase activities (RLU, relative light unit) were obtained and were calculated for REP against the maximum luciferase activity of 0.3 nM E2, which was the maximum tested concentration of E2 (E2max). In the highest concentration (5 μM) of BPA, BPS and PES, the death cell (in BPA and BPS) and chemical aggregation (in PES) were observed, thus the result in this concentration was excluded to calculate REP. The relationship of E2 was determined by comparing to E2max using the REP values (see Additional file 1: Figure S1), from which each EC_20_, EC_50_ and EC_80_ value was estimated (Table 1). The estrogenic activity of BPA increased by a dose–response manner statistically and that of BPS also was shown the increasing tendency although there was little of statistical difference (Figure 2). No estrogenic activity was observed at two low concentrations (1 and 0.1 nM) of BPA and BPS. PES showed no estrogenic activity in all of the concentration ranges tested (data was not shown). In the highest concentration of both BPA and BPS (0.5 μM), 48.8% REP of BPA and 19.2% REP of BPS were shown, respectively (Figure 2 and Table 2). By comparing these values to the respective EC values of E2, REP_20_, REP_50_ and REP_80_ of both BPA and BPS were estimated, and were summarized in Table 2. The REP_50_ of BPA and BPS was 4.25 × 10^−5^ and 4.09 × 10^−9^, respectively, and REP_20–80_ was 2.17 × 10^−4^ to 8.32 × 10^−6^ and 2.25 × 10^−6^ to 7.41 × 10^−12^, respectively.

**Table 1:** **The effective concentration (EC) values of E2, BPA and BPS**

	E2 (μM)	BPA (uM)	BPS (uM)
EC_20_	5.08 × 10^−7^	6.38 × 10^−1^	6.14 × 10^1^
EC_50_	1.05 × 10^−5^	6.70 × 10^1^	6.97 × 10^5^
EC_80_	2.18 × 10^−4^	7.04 × 10^3^	7.90 × 10^9^

**Table 2:** **Maximum concentrations of individual compounds tested in MVLN** ***in vitro*** **bioassays, induction relative to an E2 standard, and relative potency (REP) estimates**

Compounds	Max. Conc. (μM)	%-E2max ^a^	Relative potency (REP)	
			REP _50_^b^	REP _20-80_^c^
BPA	0.5	48.8	4.25 × 10^−5^	2.17 × 10^−4^ – 8.32 × 10^−6^
BPS	0.5	19.2	4.09 × 10^−9^	2.25 × 10^−6^ – 7.41 × 10^−12^
PES	0.5	-	NA^d^	

^a^%-E2max = Maximum response against the response at the highest concentration of E2 (E2max).

^b^Single point estimate of REP performed for the response of 50%-E2max.

^c^REPs reported as the range of REP estimates generated from multiple points over a response range from 20- to 80%-E2max.

^d^NA: not analyzed because the dose–response relationship was insufficient for estimation.

### Gene expression level of the estrogen receptor

**Figure 3:**
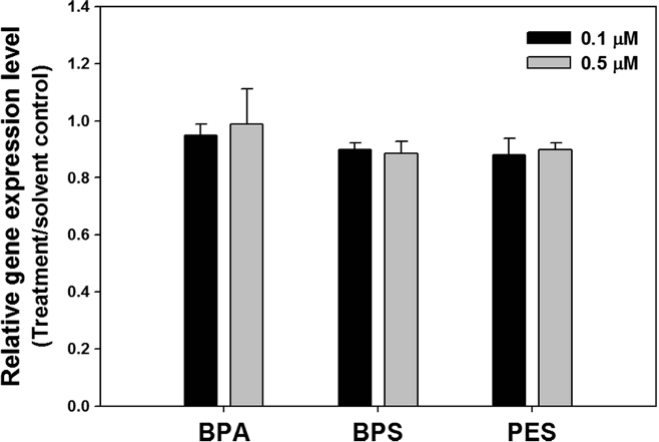
**The relative ER gene expression level induced by BPA, BPS and PES exposure in MVLN cell.** The ER gene expression level of MVLN cell treated with BPA, BPS and PES was not statistically different each other. The data were statistically analyzed by ANOVA followed by LSD test (*p* <0.05). All tests were performed in triplicate.

ER gene expression level induced by BPA, BPS and PES was compared to that by solvent (1% DMSO) (Figure 3). All of three chemicals did not affected ER gene expression level.

### Estrogenic activities of the metabolites

**Figure 4:**
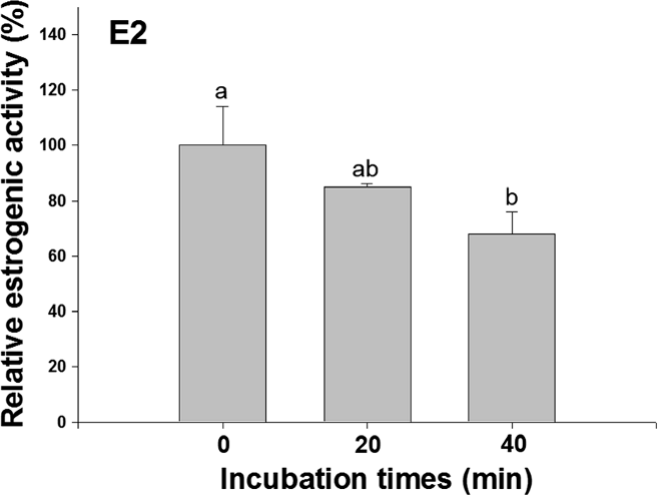
**The relative estrogenic activity of E2 metabolites compared to intact E2 incubated with an acetonitrile-denatured inactivated S9 fraction (inactive S9).** E2 (0.05 uM) was incubated with rat liver S9 for 0, 20 and 40 min at 37°C. Data represent the mean ± SD of four experiments and were statistically analyzed by ANOVA followed by LSD test (*p* <0.05). All tests were performed in triplicate.

**Figure 5:**
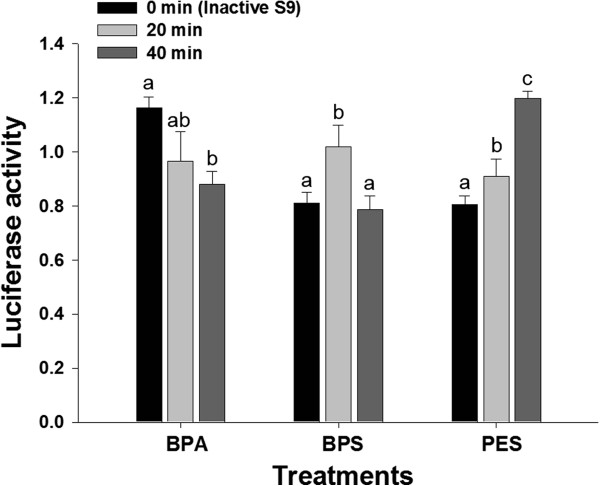
**The estrogenic activities of the metabolites of BPA, BPS and PES formed by rat liver S9 fractions according to incubation times.** The BPA, BPS and PES (0.1 mM, respectively) were incubated with rat liver S9 for 0 (inactive S9), 20 and 40 min at 37°C. The difference among incubation times of each chemical was statistically analyzed by ANOVA followed by LSD test (*p* <0.05). All tests were performed in triplicate.

The estrogenic activities of E2 metabolites according to incubation times (20 and 40 min) with an active rat liver S9 fraction were 85 and 68%, respectively, when compared to that of E2 incubated at 0 min (using an inactive rat liver S9 fraction) (Figure 4). The estrogenic activities of BPA, BPS and PES metabolites also changed according to the incubation times (Figure 5). A difference in the estrogenic activity between acetonitrile-treated PES and –untreated PES was not observed (data are not shown).

## Discussion

EDCs directly or non-directly affect the endocrine system through diverse mechanisms. Among many mechanisms, some chemicals choose an estrogenic strategy to mimic natural estrogens that directly bind to the ER and subsequently generate the unexpected endocrinal processes. Most studies have evaluated the estrogenic activity of intact chemicals and have frequently ignored the evaluation of their metabolites formed within the body. Once taken into body, the chemicals are catalyzed or metabolized by several enzymes such as oxygenases, hydrogenases and transferases. The metabolites of some estrogenic chemical by the S9 fraction showed a different level of estrogenic potency when compared to their original chemicals [17, 21, 22]. Thus, in the EDC risk assessment, the estrogenic activity of metabolites must be evaluated as well as their intact chemicals.

BPA is widely used in various industries and is a well-known estrogenic disruptor as an ER agonist but not an antagonist. BPA binds to ER, and the BPA-ER complex induces various unexpected endocrinal effects such as birth defects, reproductive, developmental, immune disorders and hormone-related cancers [29–33]. Because of its potent estrogenic disruption, the efforts to restrict its utility have been made and new BPA alternatives have been suggested. Among them, BPS has been recommended as one of BPA alternatives because it has a thermal resistance and a similar structure with BPA (Figure 1). Moreover, PES which is a polymer form of BPS linked by ether bonds has been also suggested as one of BPA alternatives (Figure 1). In the present study, we evaluated the estrogenic potency of E2, BPA, BPS and PES, and also assessed the estrogenic potential of each metabolite formed by rat liver S9 fraction.

In a primary dose–response ER binding assay, E2 showed estrogenic activity in all of the concentrations tested (Additional file 1: Figure S1). This is similar to the results of E2 estrogenic activity of other studies, indicating that the ER binding assay using the MVLN cell line in the current study was credible. Besides, the result of ER gene expression using real-time PCR supported that the estrogenic activity generated by chemical treatment was induced by ER binding but not overexpression of ER gene (Figure 3).

The estrogenicity of metabolized E2 by the rat liver S9 fraction was estimated and compared with intact E2. The estrogenic activity of E2 metabolites was decreased according to incubation times, which is in accordance with previous studies showing that E2 had a higher estrogenic activity than all of its metabolites. This result implies that the estrogenic potency of chemical can be changed by metabolism. In contrast to E2, BPA metabolites produced by the rat liver S9 fraction were known to show stronger estrogenic activity than BPA [15, 20]. Among the BPA metabolites, 4-methyl-2,4-bis (4-hydroxyphenyl) pent-1-ene (MBP) was suggested to generate estrogenic activity [17] and induced estrogenic effects in exposed organisms such as vitellogenin induction in the male medaka (*Oryzias latipes*) [34] and estrogen receptor mRNA induction in the rat [35]. In our study, however, the estrogenic activity of BPA metabolites decreased. At 60 min incubation, the estrogenic activity was shown to be comparable with 0 time (data was not shown), indicating that the estrogenic metabolites were not generated within incubation time of our study. Although our result was different from the previous studies, estrogenic studies for BPA metabolites showed that the derivatives produced by metabolism had a different estrogenicity from BPA, suggesting the requirement of an estrogenic assay for metabolites with intact chemicals.

BPS has similar physical and chemical features to BPA, but the estrogenic activity of BPS was much lower than that of BPA at tested concentration of this study (Tables 1 and 2) different from the previous study reported that the estrogenic potencies of BPA and BPS were comparable [15]. But, BPS showed the similar estrogenic activity to BPA in high concentration (see the 0 min of Figure 5). However, BPS showed dose-dependent estrogenic activity in the range of tested concentrations and the estrogenic activity of its metabolites also increased in some incubation time. This implies that BPS is not suitable as a BPA alternative. Similarly, in previous study, the estrogenic activity of the BPS metabolites that were measured using the yeast two-hybrid system and fluorescence polarization system increased after a 60 min incubation with the S9 fraction [22]. Although there is no study of metabolites of BPS formed by S9 fraction, if BPS is metabolized like BPA, the dimer of phenol with the sulfonyl group formed by metabolism of S9 fraction was supposed as the estrogenic chemical.

In dose–response assay, BPA and BPS induced estrogenic activity whereas PES did not show any estrogenic activity at the concentrations evaluated (500 to 0.1 nM). PES is a polymer form having a long length and large size (approximately 55,000 MW). Thus, it is considered that it is not easy to bind to the ER, causing little estrogenic activity. But biologically or chemically degraded PES may have an EDC potential as like BPS because PES is synthesized by the nucleophilic polycondensation of both BPS and 4,4′-dichlorodiphenylsulfone at high temperatures in the presence of potassium carbonate [18]. In current study, the estrogenic activity of the PES metabolites was observed at the ER binding assay. Interestingly, the weak estrogenic activity was also observed at high concentration (0.1 mM) of PES (in the dose response assay, the highest concentration of PES was 5 μM). Because some polar organic solvents such as acetone and acetonitrile can break the chain of PES (http://www.spectrumlabs.com/dialysis/Compatibility.html and http://www.coastpneumatics.com/chemres.html), it is not recommended that PES products be treated with these solvents [36, 37]. Since acetonitrile was used to stop the metabolism by inhibition of S9 fraction, we considered that PES treated with acetonitrile generated estrogenic activity, thus the experiment to verify the estrogenic activity of the acetonitrile-treated PES was tried. But acetonitrile-treated PES showed similar estrogenic activity to acetonitrile-untreated PES, indicating that exposure for a short time in acetonitrile did not induce the estrogenic activity of PES. Some previous studies reported that the estrogenic activity was induced by BPA released from a bottle products of the polycarbonate synthesized by a reaction of BPA and phosgene [38, 39]. Similar to BPA, the estrogenic activity of PES is suggested be caused by releasing a monomer or small sized polymer. In low concentrations, the amount of the estrogenic materials (monomer or shorter PES) was not enough to measure the estrogenic activity, but sufficient estrogenic materials seemed to present in high concentrations. During chemical treatment in the cell (72 h), the estrogenic monomer or small polymer would be released from PES and subsequently induced estrogenic activation by binding to ER. Actually, Simoneau et al. tried to detect the releases from PES on the simulant for milk 50% EtOH (as per Commission Regulation No. 321/2011 of 1 April 2011) and they certified that diphenyl sulphone was released from PES [19]. But bisphenol S was not detected. Although there is no knowledge that diphenyl sulphone is an estrogenic chemical, this result suggests the potential of releasing of estrogenic chemicals from PES on other conditions. In addition, PES metabolites showed stronger estrogenic activity compared to intact PES, indicating that PES was metabolized to other chemicals having estrogenic activity. The PES metabolites showed comparable activity to BPS metabolites. There is no knowledge of PES metabolism and its metabolites, but some natural polymers formed by ether bonds such as dextran and chitosan are known to enzymatically degrade [40], suggesting that PES is also able to cleave biologically. Our study provides the evidence that PES and PES metabolites have the estrogenic potency, which is the first report within our knowledge. Also, we hope that the estrogenic metabolites of PES will be identify in the further study.

## Conclusions

In this study, the estrogenic potencies of bisphenol derivatives (BPA, BPS and PES) and their metabolites generated by the rat liver S9 fraction were evaluated using an *in vitro* luciferase assay with transgenic MVLN cell lines. We verified the estrogenic activity of E2 and BPA and their metabolites. Also, we observed the estrogenic potency of not only BPS and PES suggested as BPA alternatives but also their metabolites. These results explain that the estrogenic activity of chemicals is able to be changed by metabolism and show the hazardous potency of BPS, PES and their metabolites as endocrine disruptors. Moreover, this study insist that the assessment of both parent chemicals and their metabolites is needed in future EDC evaluation studies.

## Electronic supplementary material

Additional file 1: Figure S1: The standard curve generated from the luciferase activity induced by E2 on MVLN cell. Data were presented as the mean values of luciferase activity induced by E2. Each data point represents the mean values of three independent experiments performed in triplicate. (TIFF 93 KB)

## References

[1] Diamanti-Kandarakis E, Bourguignon JP, Giudice LC, Hauser R, Prins GS, Soto AM, Zoeller RT, Gore AC (2009). Endocrine-disrupting chemicals: an Endocrine Society scientific statement. Endocr Rev.

[2] WHO (2002). Global Assessment of the State-of-the-Science of Endocrine Disruptors.

[3] Iavicoli I, Fontana L, Leso V, Bergamaschi A (2013). The effects of nanomaterials as endocrine disruptors. Int J Mol Sci.

[4] Erickson BE (2008). Bisphenol A under scrutiny. Chemical and Engineering News.

[5] Schwartz AW, Landrigan PJ (2012). Bisphenol A in thermal paper receipts: an opportunity for evidence-based prevention. Environ Health Perspect.

[6] Ho SM, Tang WY, Belmonte de Frausto J, Prins GS (2006). Developmental exposure to estradiol and bisphenol A increases susceptibility to prostate carcinogenesis and epigenetically regulates phosphodiesterase type 4 variant 4. Cancer Res.

[7] Markey CM, Wadia PR, Rubin BS, Sonnenschein C, Soto AM (2005). Long-term effects of fetal exposure to low doses of the xenoestrogen bisphenol-A in the female mouse genital tract. Biol Reprod.

[8] Munoz-de-Toro M, Markey CM, Wadia PR, Luque EH, Rubin BS, Sonnenschein C, Soto AM (2005). Perinatal exposure to bisphenol-A alters peripubertal mammary gland development in mice. Endocrinology.

[9] Timms BG, Howdeshell KL, Barton L, Bradley S, Richter CA, Vom Saal FS (2005). Estrogenic chemicals in plastic and oral contraceptives disrupt development of the fetal mouse prostate and urethra. Proc Natl Acad Sci U S A.

[10] Dankers AC, Roelofs MJ, Piersma AH, Sweep FC, Russel FG, Van den Berg M, Van Duursen MB, Masereeuw R (2013). Endocrine disruptors differentially target ATP-binding cassette transporters in the blood-testis barrier and affect Leydig cell testosterone secretion in vitro. Toxicol Sci.

[11] FDA (2012). Indirect Food Additives: Polymers; Food and Drug Administration.

[12] Ike M, Chen MY, Danzl E, Sei K, Fujita M (2006). Biodegradation of a variety of bisphenols under aerobic and anaerobic conditions. Water Sci Technol.

[13] Kuruto-Niwa R, Nozawa R, Miyakoshi T, Shiozawa T, Terao Y (2005). Estrogenic activity of alkylphenols, bisphenol S, and their chlorinated derivatives using a GFP expression system. Environ Toxicol Pharmacol.

[14] Zhang L, Liu LG, Pan FL, Wang DF, Pan ZJ (2012). Effects of heat treatment on the morphology and performance of PSU electrospun nanofibrous membrane. J Eng Fiber Fabr.

[15] Grignard E, Lapenna S, Bremer S (2012). Weak estrogenic transcriptional activities of Bisphenol A and Bisphenol S. Toxicol In Vitro.

[16] Vinas R, Watson CS (2013). Bisphenol S disrupts estradiol-induced nongenomic signaling in a rat pituitary cell line: effects on cell functions. Environ Health Perspect.

[17] Yoshihara S, Mizutare T, Makishima M, Suzuki N, Fujimoto N, Igarashi K, Ohta S (2004). Potent estrogenic metabolites of bisphenol A and bisphenol B formed by rat liver S9 fraction: their structures and estrogenic potency. Toxicol Sci.

[18] Colquhoun HM, Chappell D, Lewis AL, Lewis DF, Finlan GT, Williams PJ (2010). Chlorine tolerant, multilayer reverse-osmosis membranes with high permeate flux and high salt rejection. J Mater Chem.

[19] Simoneau C, Valzacchi S, Morkunas V, Van den Eede L (2011). Comparison of migration from polyethersulphone and polycarbonate baby bottles. Food Addit Contam Part A Chem Anal Control Expo Risk Assess.

[20] Kitamura S, Suzuki T, Sanoh S, Kohta R, Jinno N, Sugihara K, Yoshihara S, Fujimoto N, Watanabe H, Ohta S (2005). Comparative study of the endocrine-disrupting activity of bisphenol A and 19 related compounds. Toxicol Sci.

[21] Yoshihara S, Makishima M, Suzuki N, Ohta S (2001). Metabolic activation of bisphenol A by rat liver S9 fraction. Toxicol Sci.

[22] Hashimoto Y, Moriguchi Y, Oshima H, Kawaguchi M, Miyazaki K, Nakamura M (2001). Measurement of estrogenic activity of chemicals for the development of new dental polymers. Toxicol In Vitro.

[23] Demirpence E, Duchesne MJ, Badia E, Gagne D, Pons M (1993). MVLN cells: a bioluminescent MCE-7-derived cell line to study the modulation of estrogenic activity. J Steroid Biochem Mol Biol.

[24] Pons M, Gagne D, Nicolas JC, Mehtali M (1990). A new cellular model of response to estrogens: a bioluminescent test to characterize (anti) estrogen molecules. Biotechniques.

[25] Al-Bader M, Al-Saji S, Ford CH, Francis I, Al-Ayadhy B (2010). Real-time PCR: detection of oestrogen receptor-alpha and -beta isoforms and variants in breast cancer. Anticancer Res.

[26] Kurebayashi J, Otsuki T, Kunisue H, Tanaka K, Yamamoto S, Sonoo H (2000). Expression levels of estrogen receptor-alpha, estrogen receptor-beta, coactivators, and corepressors in breast cancer. Clin Cancer Res.

[27] Livak KJ, Schmittgen TD (2001). Analysis of relative gene expression data using real-time quantitative PCR and the 2(−Delta Delta C(T)) Method. Methods.

[28] Villeneuve DL, Khim JS, Kannan K, Giesy JP (2002). Relative potencies of individual polycyclic aromatic hydrocarbons to induce dioxinlike and estrogenic responses in three cell lines. Environ Toxicol.

[29] Kubo K, Arai O, Omura M, Watanabe R, Ogata R, Aou S (2003). Low dose effects of bisphenol A on sexual differentiation of the brain and behavior in rats. Neurosci Res.

[30] Maffini MV, Rubin BS, Sonnenschein C, Soto AM (2006). Endocrine disruptors and reproductive health: the case of bisphenol-A. Mol Cell Endocrinol.

[31] Roy A, Bauer SM, Lawrence BP (2012). Developmental exposure to bisphenol A modulates innate but not adaptive immune responses to influenza A virus infection. PLoS One.

[32] Vandenberg LN, Maffini MV, Wadia PR, Sonnenschein C, Rubin BS, Soto AM (2007). Exposure to environmentally relevant doses of the xenoestrogen bisphenol-A alters development of the fetal mouse mammary gland. Endocrinology.

[33] Weber Lozada K, Keri RA (2011). Bisphenol A increases mammary cancer risk in two distinct mouse models of breast cancer. Biol Reprod.

[34] Ishibashi H, Watanabe N, Matsumura N, Hirano M, Nagao Y, Shiratsuchi H, Kohra S, Yoshihara S, Arizono K (2005). Toxicity to early life stages and an estrogenic effect of a bisphenol A metabolite, 4-methyl-2,4-bis(4-hydroxyphenyl)pent-1-ene on the medaka (Oryzias latipes). Life Sci.

[35] Okuda K, Takiguchi M, Yoshihara S (2010). In vivo estrogenic potential of 4-methyl-2,4-bis(4-hydroxyphenyl)pent-1-ene, an active metabolite of bisphenol A, in uterus of ovariectomized rat. Toxicol Lett.

[36] **Chemical Compatibility** [http://www.spectrumlabs.com/dialysis/Compatibility.html]

[37] **Chemical Resistance of Polypulfone** [http://www.coastpneumatics.com/chemres.html]

[38] Krishnan AV, Stathis P, Permuth SF, Tokes L, Feldman D (1993). Bisphenol-A: an estrogenic substance is released from polycarbonate flasks during autoclaving. Endocrinology.

[39] Le HH, Carlson EM, Chua JP, Belcher SM (2008). Bisphenol A is released from polycarbonate drinking bottles and mimics the neurotoxic actions of estrogen in developing cerebellar neurons. Toxicol Lett.

[40] Markovsky E, Baabur-Cohen H, Eldar-Boock A, Omer L, Tiram G, Ferber S, Ofek P, Polyak D, Scomparin A, Satchi-Fainaro R (2012). Administration, distribution, metabolism and elimination of polymer therapeutics. J Control Release.

