# Supplementary figures and images for: Estrogenic potency of bisphenol S, polyethersulfone and their metabolites generated by the rat liver S9 fractions on a MVLN cell using a luciferase reporter gene assay
Source: Reprod Biol Endocrinol. 2014 Nov 4;12:102. doi: 10.1186/1477-7827-12-102 (PMC4232735; doi:10.1186/1477-7827-12-102)

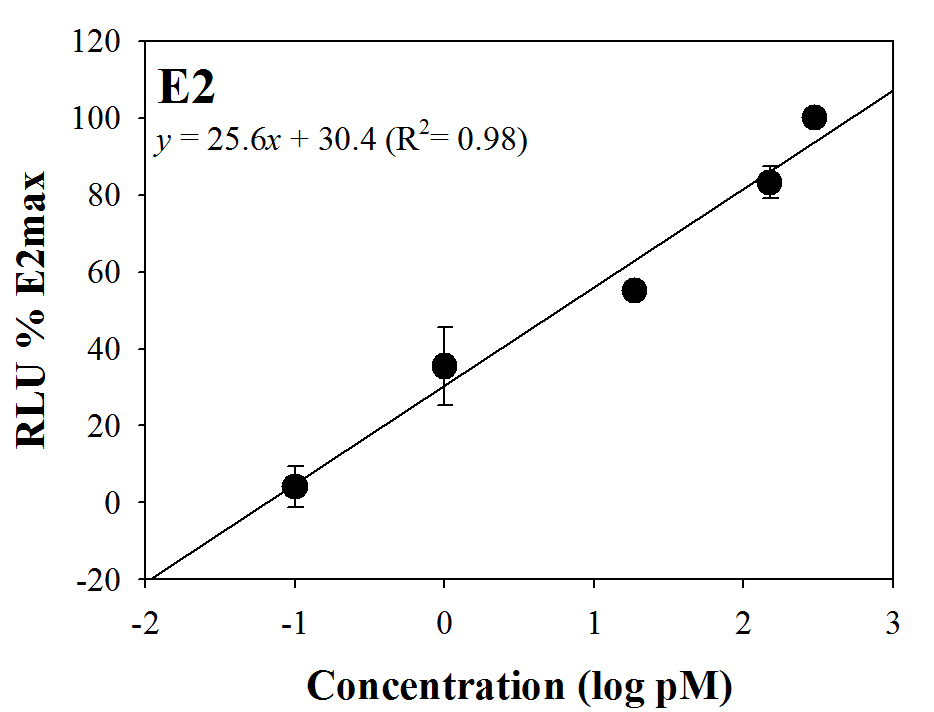

Supplement: Supplementary file 1 — Additional file 1: Figure S1: The standard curve generated from the luciferase activity induced by E2 on MVLN cell. Data were presented as the mean values of luciferase activity induced by E2. Each data point represents the mean values of three independent experiments performed in triplicate. (TIFF 93 KB) [file 12958_2014_1271_MOESM1_ESM.tiff]
